# Development and Validation of the Japanese Version of the Hyperhidrosis Quality of Life Index

**DOI:** 10.1111/1346-8138.70101

**Published:** 2025-12-10

**Authors:** Sayaka Ogawa, Jun Tayama, Paul Kamudoni, Sam Salek, Peter Bernick, Masakazu Kobayashi, Seiko Nakamichi, Hiroyuki Murota

**Affiliations:** ^1^ Faculty of Humanities Nagasaki Junshin Catholic University Nagasaki Japan; ^2^ Faculty of Human Sciences Waseda University Tokorozawa Japan; ^3^ Merck Healthcare KGaA Darmstadt Germany; ^4^ School of Life and Medical Sciences University of Hertfordshire Hertfordshire UK; ^5^ Student Accessibility Office Nagasaki University Nagasaki Japan; ^6^ Health Center Nagasaki University Nagasaki Japan; ^7^ Department of Dermatology Nagasaki University Nagasaki Japan

**Keywords:** HidroQoL, hyperhidrosis, quality of life, reliability, validity

## Abstract

Hyperhidrosis decreases an individual's quality of life (QOL). The Hyperhidrosis Quality of Life Index (HidroQoL) measures the impact of hyperhidrosis on QOL and has established reliability and validity. However, a Japanese version does not exist. Hence, this study aimed to develop the Japanese version of the HidroQoL (HidroQoL‐J) and verify its reliability and validity. The first survey included 528 participants (272 males, 256 females, mean age ± standard deviation 41.89 ± 15.24 years) who met the criteria for hyperhidrosis and scored ≥ 2 on the Hyperhidrosis Disease Severity Scale (HDSS). The second survey was conducted for reliability and included 210 participants (105 males, 105 females, mean age ± standard deviation 43.56 ± 14.60 years). The main survey items were (1) HidroQoL‐J, (2) Dermatology Life Quality Index (DLQI), (3) Skindex‐16, and (4) Anxiety Scale Specific to Hyperhidrosis Symptoms (ASSHS). Confirmatory factor analysis revealed the HidroQoL‐J had a two‐factor structure: a “daily life activities domain” with six items and a “psychosocial life domain” with 12 items, as in the original English version of the instrument. Cronbach's alphas (*α*) for the HidroQoL‐J were 0.93, 0.85, and 0.91 for overall, daily life activities, and psychosocial life, respectively. Test–retest reliability was *r* = 0.70, 0.67, and 0.69 for overall, daily life activities, and psychosocial life (all *p* < 0.001), respectively. Furthermore, the intraclass correlation coefficients were 0.70, 0.67, and 0.69, respectively. Moderate positive correlations were observed between the overall HidroQoL‐J score and the DLQI (*r* = 0.56) and Skindex‐16 (*r* = 0.43) scores (all *p* < 0.001). There was also a moderate positive correlation between the overall score of the HidroQoL‐J and HDSS (*r* = 0.42) and a weak positive correlation with ASSHS (*r* = 0.39) (all *p* < 0.001). Therefore, the HidroQoL‐J exhibited sufficient reliability and validity to measure the impact of hyperhidrosis symptoms on QOL.

## 1 Introduction

Primary focal hyperhidrosis (hyperhidrosis) is a chronic skin disorder characterized by bilateral excessive sweating localized on the palmar, plantar, axillary or head/face sites [1, 2, 3]. Prevalence in Japan has been reported to be approximately 10.0% [2].

Hyperhidrosis affects various aspects of daily life, such as schoolwork, work, and relationships, and decreases quality of life (QOL). Previous studies have reported that patients with hyperhidrosis have a lower skin‐related QOL compared with healthy individuals [4, 5]. A study reported reduced QOL in 85.5% of 24 patients with axillary hyperhidrosis and high scores on the Hyperhidrosis Severity Scale [6]. In a Brazilian survey of 1658 patients with hyperhidrosis, 94% reported poor or very poor QOL [7]. A Danish study reported that patients with hyperhidrosis (*n* = 2794) had lower mental and physical health‐related QOL than those without hyperhidrosis (*n* = 20 458), even after adjusting for confounding factors, such as sex, age, lifestyle, socioeconomic status, stress, region, season, and other comorbidities [8]. Furthermore, a systematic review and meta‐analysis of 11 eligible studies on the association between hyperhidrosis and QOL reported that patients with hyperhidrosis had higher Dermatology Life Quality Index (DLQI) scores and lower health‐related QOL compared to those without hyperhidrosis [9].

Hyperhidrosis symptoms reduce patients' quality of life in both social and psychological domains. A systematic review of 49 studies on the impact of hyperhidrosis on QOL revealed that patients with hyperhidrosis reported decreased well‐being and increased anxiety and depression, as well as functional, social, occupational, and physical impairments [10]. Studies in Japan have also indicated that hyperhidrosis exerts diverse effects on quality of life. In a survey of 608 Japanese patients with axillary hyperhidrosis who reported its effects on their daily lives, 17.1% reported it impacted their school or work [11]. Furthermore, in a cross‐sectional study on 321 patients with axillary hyperhidrosis 30.5% of participants reported decreased productivity, with decreases tending to be greater in those more severely affected [12].

The DLQI, which measures skin‐related quality of life, 36‐item Short Form (SF‐36), and Skindex‐16 are often used to measure QOL in patients with hyperhidrosis. A German study utilized the DLQI and revealed that QOL impairment in patients with hyperhidrosis was equal to or greater than that in patients with skin diseases, such as psoriasis, atopic dermatitis, urticaria, and acne [4]. A cross‐sectional study of 4500 patients from an outpatient clinic in Jordan reported that the mean DLQI score for 144 patients with hyperhidrosis was 12.8 points (indicating major QOL impairment), while 31.9% and 59.1% of these 144 patients had moderate and severe QOL impairment, respectively [13]. A Swedish study of 95 hyperhidrosis patients found that the mean DLQI score was 15.8, indicating a significant impact on quality of life [14]. In addition, the SF‐36 was used to measure health‐related quality of life in 160 patients with hyperhidrosis, and revealed lower mental health scores in patients with axillary, plantar, and palmar hyperhidrosis [15]. The Skindex‐16, a scale with three factors: “symptoms” (degree of itching and stinging), “emotions” (degree of embarrassment, depression), and “functioning” (interference with daily life and social interaction), has been used to measure quality of life in hyperhidrosis patients. The previously mentioned Swedish cross‐sectional study compared previously reported Skindex‐16 values for psoriasis, eczema, acne, and hyperhidrosis [14]. Results indicated that individuals with hyperhidrosis had lower symptom factor scores on the Skindex‐16 than those with psoriasis, eczema, and acne. However, patients with hyperhidrosis had higher scores on the emotional factor of the Skindex‐16, similar to patients with psoriasis, and even higher than those with eczema and acne [14]. Regarding the functioning factor of the Skindex‐16, patients with hyperhidrosis had higher scores than those with psoriasis, eczema, and acne, and high scores in the emotional and functioning factors, which indicated poor QOL [14].

The DLQI, SF‐36, and Skindex‐16 have been used to measure QOL in patients with hyperhidrosis. However, the DLQI assesses the impact of skin conditions on QOL and may not fully capture the effects of hyperhidrosis symptoms on QOL [16]. Therefore, Kamudoni et al. [17] created a disease‐specific quality of life scale for hyperhidrosis (Hyperhidrosis Quality of Life Index: HidroQoL) to understand its impact on QOL in detail. The reliability and validity of the HidroQoL have been confirmed to be high [17]. This scale assesses quality of life by dividing it into daily life activities (6 items) and psychosocial life (12 items), enabling detailed evaluation of hyperhidrosis symptoms' impact on everyday functioning [17]. Gabes et al. [18] conducted a systematic review of the quality of existing “patient‐reported outcome measures (PROMs),” validated for hyperhidrosis via the COnsensus‐based Standards for the selection of health Measurement INstruments (COSMIN) method. They reported that HidroQoL could be recommended as a PROM for use in future clinical trials of hyperhidrosis [18]. However, a Japanese version of the HidroQoL (HidroQoL‐J) has not been created. Its development will enable accurate assessment of the QOL of patients with hyperhidrosis in Japan and may be useful in verifying the effectiveness of hyperhidrosis treatment. It can also lead to a better understanding of the psychological aspects of patients with hyperhidrosis. Therefore, this study aimed to develop a HidroQoL‐J and verify its reliability and validity.

## 2 Methods

### 2.1 Development of the HidroQoL‐J

Permission to culturally adapt the HidroQoL into Japanese was obtained from the author of the original HidroQoL. The original HidroQoL [17] was distributed under a Creative Commons Attribution License. This license permits use, distribution, and modification for non‐commercial academic and clinical purposes, provided that the original authors and sources are properly cited. Therefore, the HidroQoL‐J can also be used under the same conditions.

The HidroQoL was translated into Japanese via standardized procedures. Two researchers independently translated the original text, after which a dermatologist, who specialized in hyperhidrosis, reviewed the Japanese translations for consistency. A bilingual researcher, whose native language was English, back‐translated the HidroQoL‐J into English. Comparison between the back‐translated and original versions demonstrated agreement among the researchers, who included the original author, Kamudoni, regarding the nuances of the HidroQoL. Consequently, the HidroQoL‐J (18 items across two domains) was developed (Table 1 and Table S1).

**TABLE 1:** Original version of the HidroQoL [17].

No		Very much	A little	No, not at all
**Domain 1: Daily life activities**				
1	My choice of clothing is affected	2	1	0
2	My physical activities are affected	2	1	0
3	My hobbies are affected	2	1	0
4	My work is affected	2	1	0
5	I worry about the additional activities in dealing with my condition	2	1	0
6	My holidays are affected (e.g., planning, activities)	2	1	0
**Domain 2: Psychosocial life**				
7	I feel nervous	2	1	0
8	I feel embarrassed	2	1	0
9	I feel frustrated	2	1	0
10	I feel uncomfortable physically expressing affection (e.g., hugging)	2	1	0
11	I think about sweating	2	1	0
12	I worry about my future health	2	1	0
13	I worry about people's reactions	2	1	0
14	I worry about leaving sweat marks on things	2	1	0
15	I avoid meeting new people	2	1	0
16	I avoid public speaking (e.g., presentations)	2	1	0
17	My appearance is affected	2	1	0
18	My sex life is affected	2	1	0

### 2.2 Study Participants

This study followed Kamudoni et al.'s study [17] and recruited participants who ranged from teenagers to individuals in their 60s. Teenage participants were recruited through a class taught by the first author and invited to participate. For participants in their 20s–60s, recruitment was outsourced to a web survey company, Cross Marketing Inc.; those who participated received a nominal amount of compensation to cover telecommunication costs.

Inclusion criteria were (1) Japanese individuals aged 18 years or older, (2) diagnosed with hyperhidrosis according to the diagnostic criteria [19], and (3) had a severity rating of ≥ 2 on the Hyperhidrosis Disease Severity Scale (HDSS) [20]. Of the 565 individuals who completed the survey, 37 were excluded: 23 due to suspected secondary hyperhidrosis, three due to generalized hyperhidrosis, three due to identifying their sex as other, and eight due to having an HDSS score of 1. Hence, 528 (272 males and 256 females, mean age ± standard deviation 41.89 ± 15.24 years) were included. Furthermore, according to the COSMIN guidelines, a sufficient sample size should be seven times the number of items in the scale and include ≥ 100 participants when examining construct validity for PROMs [21, 22]. This study met these criteria, indicating an adequate sample size.

To examine the test–retest reliability of the HidroQoL‐J, participants in the first survey who provided consent for the second survey were resurveyed 3 weeks later. The second survey included 210 participants (105 males and 105 females, mean age ± standard deviation 43.56 ± 14.60 years).

### 2.3 Study Procedure

This study used a web‐based survey method. The first survey “Survey on sweat and quality of life” was conducted in November 2023 and January 2024. A second survey followed 3 weeks later in December 2023 and February 2024 to verify reliability. In both surveys, a document explained the purpose, voluntary nature, and protection of personal information on the first page, and participants' cooperation was requested. Respondents could “agree” or “disagree” to cooperate, and checking “agree” indicated their consent.

### 2.4 Measurements

#### 2.4.1 HidroQoL‐J Translated in This Study

The HidroQoL‐J used the original 18‐item HidroQoL developed by Kamudoni et al. [17] translated into Japanese. It measured hyperhidrosis impact on QOL, comprising 18 items: six in daily life activities and 12 in psychosocial life domains. Each item was rated on a 3‐point Likert scale from 0 to 2, with scores ranging 0–36 points. A higher HidroQoL score indicated a greater negative impact of hyperhidrosis on quality of life.

#### 2.4.2 Diagnostic Criteria for Hyperhidrosis [19] and Site of High Sweating

Hornberger et al.'s diagnostic criteria for hyperhidrosis were used [19]. Participants were asked, “Do you have excessive sweating on the head/face, palmar, Planter of the feet, and axillary for over six months without apparent cause?” Those answering “yes” were asked: (1) Were you under 25 years old when the symptoms first appeared? (2) Do you have symmetrical sweating? (3) Does sweating stop during sleep? (4) Do you have at least one hyperhidrosis episode per week? (5) Do you have family members with hyperhidrosis? and (6) Does sweating interfere with daily life? Respondents answering “yes” to at least two questions were judged hyperhidrosis symptomatic.

Regarding their high sweating site, participants were asked, “Which of the following sites causes the most sweating: head/face, palmar, plantar, axillary, or other sites? Please select one item that is applicable.”

#### 2.4.3 Severity of Hyperhidrosis [20]

The HDSS by Strutton et al. was used to determine the severity of hyperhidrosis. Participants selected one of the four provided options that most closely represented their subjective symptoms and frequency of sweating: (1) “My sweating is never noticeable and never interferes with my daily activities,” (2) “My sweating is tolerable but sometimes interferes with my daily activities,” (3) “My sweating is barely tolerable and frequently interferes with my daily activities,” or (4) “My sweating is intolerable and always interferes with my daily activities.”

#### 2.4.4 Japanese Version of Dermatology Life Quality Index (DLQI) [23]

The DLQI measured health‐related QOL related to skin diseases. It comprised 10 items rated on a 4‐point Likert scale that ranged from 0 to 3. Higher scores indicated poorer health‐related QOL related to skin diseases [23].

#### 2.4.5 Japanese Version of Skindex‐16 [24]

The Japanese version of the Skindex‐16 developed by Higaki et al. [24] was used. It measured the impact of skin diseases QOL and comprised 16 items and three factors: symptoms (four items), emotions (seven items), and functioning (five items). Each item was scored on a 7‐point Likert scale that ranged from 0 (never) to 6 (always). The scores are converted to a linear scale ranging from 0 to 100; the higher the scores, the poorer the respondent's skin diseases QOL.

#### 2.4.6 Anxiety Scale Specific to Hyperhidrosis Symptoms (ASSHS) [25]

We used the ASSHS developed by Ogawa et al. [25] It comprised 10 items, and respondents answered each item on a 5‐point Likert scale (0 = not at all applicable to 4 = very applicable). Higher scores indicated higher levels of anxiety specific to hyperhidrosis symptoms.

#### 2.4.7 Sociodemographic Data

We enquired participants' sex, age, duration of hyperhidrosis symptoms, employment status (1 employed, 2 unemployed, 3 retired, 4 student), and nationality (Japanese, other).

### 2.5 Statistical Analysis

A confirmatory factor analysis (CFA) was conducted based on the factor structure of the original HidroQoL, and the goodness of fit of the two‐factor model of the HidroQoL‐J was calculated. To confirm the reproducibility of the established factor structure of the original HidroQoL, a CFA was conducted based on methods reported in previous studies [26, 27, 28], which similarly developed Japanese versions of scales from their original versions. We calculated the chi‐square (*χ* ^2^), goodness‐of‐fit index (GFI), adjusted goodness‐of‐fit index (AGFI), comparative fit index (CFI), and root mean square error of approximation (RMSEA) values as goodness‐of‐fit indices.

Cronbach's alpha, Pearson's product–moment correlation coefficients, and intraclass correlation coefficients (ICCs) were used to evaluate the reliability of the HidroQoL‐J. ICCs were calculated via a two‐way random‐effects model with absolute agreement and single measurements for test–retest reliability. Furthermore, to examine construct validity, Pearson's product‐moment correlation coefficients were calculated for the HidroQoL‐J and DLQI, Skindex‐16 (overall, symptoms, emotions, and functioning), HDSS, and ASSHS.

Statistical significance was set at *p* < 0.05 (two‐tailed). Analyses were performed via IBM SPSS Statistics version 28 and Amos version 28 (IBM Japan Inc., Tokyo, Japan).

### 2.6 Ethical Considerations

This study was approved by Nagasaki Junshin Catholic University Graduate School of Humanistic Studies' ethics committee (2023011A). The survey's first page presented the study purpose, method, privacy considerations, voluntary participation, and that declining would cause no disadvantage. Participants could “agree” or “disagree.” Consent was obtained when respondents checked “agree.” This study followed the Declaration of Helsinki and Ethical Guidelines for Medical Research Involving Human Subjects.

## 3 Results

### 3.1 Participants' Demographic Data

Table 2 presents participants' demographic data. The first survey included 528 participants: 272 males (51.52%) and 256 females (48.48%), with a mean age of 41.89 (standard deviation 15.24) years. The most common duration of hyperhidrosis symptoms was ≥ 10 years (348 participants, 65.91%), with the most commonly affected site being the palmar (32.19%). Hyperhidrosis severity was categorized as: HDSS 2 (67.80%), 3 (22.73%), and 4 (9.47%). Regarding employment status, the majority of participants were employed (68.37%).

**TABLE 2:** Participants' demographic data.

	First survey (*n* = 528)
Sex, *n* (%)	
Male	272 (51.52%)
Female	256 (48.48%)
Age mean (standard deviation)	41.89 (15.24)
Duration of condition, years, *n* (%)	
< 1 year	3 (0.57%)
1 to < 5 years	91 (17.23%)
5 to < 10 years	86 (16.29%)
> 10 years	348 (65.91%)
Site of high sweating, *n* (%)	
Palmar	170 (32.19%)
Plantar	129 (24.43%)
Axillary	56 (10.61%)
Head/Face	156 (29.55%)
Other	17 (3.22%)
HDSS, *n* (%)	
HDSS 2	358 (67.80%)
HDSS 3	120 (22.73%)
HDSS 4	50 (9.47%)
Employment status, *n* (%)	
Employed	361 (68.37%)
Unemployed	87 (16.48%)
Retired	12 (2.27%)
Full‐time student	68 (12.88%)
HidroQoL‐J Overall mean (standard deviation)	18.54 (9.08)
Daily life activities mean (standard deviation)	5.68 (3.34)
Psychosocial life mean (standard deviation)	12.87 (6.31)
DLQI mean (standard deviation)	6.41 (7.79)
Skindex‐16 Overall mean (standard deviation)	28.68 (23.30)
Skindex‐16 Symptoms mean (standard deviation)	24.61 (23.79)
Skindex‐16 Emotions mean (standard deviation)	32.54 (25.78)
Skindex‐16 Functioning mean (standard deviation)	26.55 (25.37)
ASSHS mean (standard deviation)	23.27 (9.83)

Abbreviations: ASSHS, anxiety scale specific to hyperhidrosis symptoms; DLQI, dermatology life quality index; HDSS, hyperhidrosis disease severity scale.

The mean total score for the HidroQoL‐J was 18.54, with subscale means of 5.68 for daily life activities and 12.87 for psychosocial life. The mean scores for the other measures were 6.41 for the DLQI, 28.68 for the Skindex‐16, and 23.27 for the ASSHS.

### 3.2 Confirmatory Factor Analysis

**FIGURE 1:**
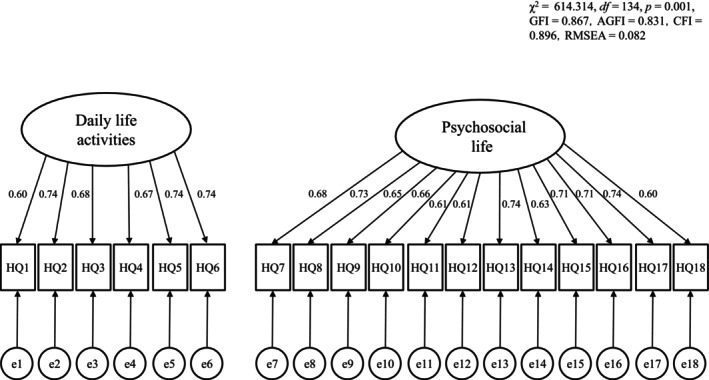
Results of the covariance structural analysis.

CFA of the HidroQoL‐J 18‐item two‐factor model was conducted via data from the first survey. Results revealed *χ* ^2^ = 614.314, df = 134, *p* = 0.001, GFI = 0.867, AGFI = 0.831, CFI = 0.896, and RMSEA = 0.082. RMSEA was not a good indicator of model fit. However, the other indicators had acceptable values (Figure 1).

### 3.3 Consideration of Reliability

Cronbach's alpha coefficients for the HidroQoL‐J in the first survey were 0.93 for the overall scale, 0.85 for the daily life activities domain, and 0.91 for the psychosocial life domain. A strong positive correlation was also observed between the daily life activities and psychosocial life domains of the HidroQoL‐J (*r* = 0.85, *p* < 0.001).

For test–retest reliability, correlation coefficients between the first and second surveys 3 weeks later were calculated: overall *r* = 0.70 (*p* < 0.001), daily life activities domain *r* = 0.67 (*p* < 0.001), and psychosocial life domain *r* = 0.69 (*p* < 0.001). Furthermore, to evaluate the test–retest reliability, the ICC was calculated, showing values of 0.70 (95% CI: 0.63–0.76) for the overall scale, 0.67 (95% CI: 0.59–0.74) for the daily life activities domain, and 0.69 (95% CI: 0.62–0.76) for the psychosocial life domain.

### 3.4 Consideration of Validity

Table 3 presents the correlation coefficients between the HidroQoL‐J and each item. The DLQI and Skindex‐16 were used to assess criterion‐related validity, while the HDSS and ASSHS were used to examine construct validity.

**TABLE 3:** HidroQoL‐J and correlation of each item.

	HidroQoL‐J	HidroQoL‐J	HidroQoL‐J
	Overall	Daily life activities	Psychosocial life
DLQI	0.56*	0.58*	0.49*
Skindex‐16 Overall	0.43*	0.44*	0.38*
Skindex‐16 Symptoms	0.32*	0.36*	0.27*
Skindex‐16 Emotions	0.38*	0.38*	0.34*
Skindex‐16 Functioning	0.48*	0.47*	0.45*
HDSS	0.42*	0.44*	0.36*
ASSHS	0.39*	0.27*	0.41*

Abbreviations: ASSHS, anxiety scale specific to hyperhidrosis symptoms; DLQI, dermatology life quality index; HDSS, hyperhidrosis disease severity scale.

* *p* < 0.001.

#### 3.4.1 Criterion‐Related Validity

Moderate positive correlations were observed between the overall score of the HidroQoL‐J and the DLQI (*r* = 0.56), the overall score of the Skindex‐16 (*r* = 0.43), and Skindex‐16 functioning (*r* = 0.48) (all *p* < 0.001). Weak positive correlations were also observed with Skindex‐16 symptoms (*r* = 0.32) and Skindex‐16 emotions (*r* = 0.38) (all *p* < 0.001). Similarly, moderate positive correlations were found between the daily life activities domain and the DLQI (*r* = 0.58), the overall score of the Skindex‐16 (*r* = 0.44), and Skindex‐16 functioning (*r* = 0.47) (all *p* < 0.001). Moderate positive correlations were observed between the psychosocial life domains and the DLQI (*r* = 0.49) and Skindex‐16 functioning (*r* = 0.45) (all *p* < 0.001).

#### 3.4.2 Construct Validity

A moderate positive correlation was observed between the overall score of the HidroQoL‐J and the HDSS, which measured the severity of hyperhidrosis (*r* = 0.42, *p* < 0.001). A weak positive correlation was also observed between the overall score of the HidroQoL‐J and the ASSHS (*r* = 0.39, *p* < 0.001).

In addition, a moderate positive correlation was observed between the daily life activities domain of the HidroQoL‐J and the HDSS (*r* = 0.44, *p* < 0.001), while a weak positive correlation was observed with the ASSHS (*r* = 0.27, *p* < 0.001).

A moderate positive correlation was also observed between the psychosocial life domains of the HidroQoL‐J and the ASSHS (*r* = 0.41, *p* < 0.001), whereas a weak positive correlation was found with the HDSS (*r* = 0.36, *p* < 0.001).

## 4 Discussion

This study developed the HidroQoL‐J and verified its reliability and validity. The HidroQoL‐J had a two‐factor structure: “daily life activities” and “psychosocial life” domains with six and 12 items respectively, similar to Kamudoni et al.'s original study [17]. Strong positive correlations were confirmed between the HidroQoL‐J and both domains. The HidroQoL‐J showed good internal consistency and test–retest reliability. Significant correlations were observed between the HidroQoL‐J and the DLQI, Skindex‐16, HDSS, and ASSHS. Hence, the reliability and validity of the HidroQoL‐J were confirmed in this study, with acceptable levels of internal consistency and test–retest reliability.

### 4.1 Factorial Validity and Reliability of the HidroQoL‐J

Results of the CFA suggested that the model's goodness‐of‐fit indices were sufficient, with the exception of the RMSEA, which had acceptable values. The HidroQoL‐J had a two‐factor structure with “daily life activities domain” and “psychosocial life domain” as in Kamudoni et al. [17] Donhauser et al. [29] examined 529 patients with severe axillary hyperhidrosis with HDSS scores of 3–4 who responded to the HidroQoL. The HidroQoL has been reported as a unidimensional scale that produces a single overall score while showing a two‐factor structure for calculating separate domain scores for daily life activities and psychosocial life [29]. The HidroQoL‐J demonstrated a two‐factor structure, and the high correlation between the factors suggests that it can also be used as a unidimensional scale by calculating an overall score.

Cronbach's alpha coefficients were calculated for the HidroQoL‐J as overall, daily life activities domain, and psychosocial life domain. All values were high, indicating strong internal consistency. The Cronbach's alpha coefficient reported by Kamudoni et al. [17] was comparable to this study's value. In addition, the high correlation coefficients between the HidroQoL‐J overall, daily life activities domain, and psychosocial life domain in the first and second surveys suggested that it had sufficient reliability as a measurement scale. Furthermore, ICC results supported the reproducibility of the HidroQoL‐J as a measurement scale.

### 4.2 Criterion‐Related Validity of the HidroQoL‐J

Moderate positive correlations were observed between the overall score of the HidroQoL‐J and the DLQI, the overall score of Skindex‐16, and Skindex‐16 functioning, which assess QOL specific to skin diseases. These findings indicate that the HidroQoL‐J reflects skin disease–related QOL, supporting its criterion‐related validity. The original HidroQoL [17] also showed a moderate correlation with the DLQI, consistent with prior findings. However, the HidroQoL‐J showed only weak positive correlations with the Skindex‐16 symptoms and emotions subscales. Since the HidroQoL measures the impact of hyperhidrosis on daily life, psychosocial impact, and broader disease effects [17], it is considered that the correlation with the HidroQoL‐J was weak, as measuring the symptom and emotional aspects of the Skindex‐16 focus on specific domains.

### 4.3 Construct Validity of the HidroQoL‐J

A moderate positive correlation was observed between the overall score of the HidroQoL‐J and HDSS, which measures hyperhidrosis severity. This suggests the HidroQoL‐J reflects hyperhidrosis symptoms, confirming acceptable construct validity. Moderate positive correlations were also observed between the original HidroQoL [17] and HDSS and between the Arabic version [30] and HDSS, consistent with prior studies. A weak positive correlation was observed between the overall score of the HidroQoL‐J and ASSHS. The ASSHS differs from HidroQoL, which assesses overall QOL, likely explaining the weaker correlation.

Patient‐reported outcome measures (PROMs) effectively evaluate QOL in patients with hyperhidrosis [10]. Their benefits include improved patient‐provider communication, precise outcome measurement, and better understanding of treatment impact [31]. The HidroQoL is a PROM offering ease of use and clinical applicability [32]. Given its comparable structure, reliability, and validity, the HidroQoL‐J appears suitable for clinical use in Japan.

### 4.4 Limitations and Future Challenges

This study has several limitations. First, participants self‐reported hyperhidrosis based on Hornberger et al.'s diagnostic criteria [19]. Since diagnosis was not made by a physician, caution should be exercised when interpreting results. Future studies should examine whether the same results can be obtained in patients diagnosed with hyperhidrosis by physicians at medical institutions. Second, this survey was conducted via the internet, so only those with internet access responded. Hence, future studies should include paper‐based questionnaires and face‐to‐face surveys. Third, patients with generalized hyperhidrosis were not included. Kamudoni et al. [17] also included generalized hyperhidrosis; however, this study was limited to primary focal hyperhidrosis owing to different pathogenic mechanisms and influencing factors. Future research should conduct surveys of patients with generalized hyperhidrosis via the HidroQoL‐J. Fourth, surveys for teenagers and adults in their 20s to 60s used different methods. For teenagers, the survey was distributed to university students in a class taught by the first author. For participants in their 20s to 60s, the survey was administered by an online survey company. Since both groups met the selection criteria of hyperhidrosis diagnosis and HDSS score of 2 or higher, homogeneity was generally ensured; however future surveys should use a consistent method across age groups.

In a study of patients with severe axillary hyperhidrosis (HDSS 3–4), 87 patients using glycopyrronium bromide 1% cream showed a significant decrease in sweat volume, severity, and HidroQoL scores after 29 days compared with 84 patients in the placebo group [33]. To assess QOL decline in patients with hyperhidrosis in Japan and consider treatment plans, examining effects of hyperhidrosis severity and sweating site on QOL using the HidroQoL‐J is necessary. Further it is considered necessary for future studies to incorporate cognitive debriefing interviews with Japanese patients with hyperhidrosis to ensure that the translated items are interpreted as intended and are culturally appropriate.

## 5 Conclusions

We developed the HidroQoL‐J and examined its reliability and validity. The HidroQoL‐J had the same two‐factor structure of “daily life activities domain” and “psychosocial life domain” as the original version of the HidroQoL. Furthermore, the HidroQoL‐J demonstrated sufficient reliability and validity as a scale for measuring the impact of hyperhidrosis symptoms on QOL.

## Funding

This work was supported by the MHLW Research Program on Rare and Intractable Diseases, Grant Number JPMH25FC1014, and by JSPS KAKENHI, Grant Number JP24K06612.

## Ethics Statement

The study was conducted in accordance with the Declaration of Helsinki and approved by the Ethics Committee of the Nagasaki Junshin Catholic University Graduate School of Humanistic Studies (approval number: 2023011A). Informed consent was obtained online by having participants read the study details and select “agree” before proceeding to the survey. Animal Studies: N/A.

## Conflicts of Interest

S.O. has received consulting fees and/or speaker honoraria from Maruho Co. Ltd., Kaken Pharmaceutical Co. Ltd., and Hisamitsu Pharmaceutical Co. Inc. H.M. has received consulting fees and/or speaker honoraria from Kaken Pharmaceutical Co. Ltd., Maruho Co. Ltd., and Hisamitsu Pharmaceutical Co. Inc. P.K. is a joint copyright holder of the HidroQoL and is an employee of Merck Healthcare KGaA. Merck does not currently have a product for the treatment of hyperhidrosis. S.S. is a joint copyright holder of the HidroQoL, and has received educational grants from Bristol Meyer Squibb, European Hematology Association, GlaxoSmithkline, Novartis and Sanofi.

## Supporting information


**Table S1:** The Japanese version of the HidroQoL (HidroQoL‐J).

## Data Availability

The data that support the findings of this study are available from the corresponding author upon reasonable request.
