# Supplementary material for: Development and Validation of the Japanese Version of the Hyperhidrosis Quality of Life Index
Source: J Dermatol. 2025 Dec 10;53(2):210–8. doi: 10.1111/1346-8138.70101 (PMC12877979; doi:10.1111/1346-8138.70101)
Supplement: Supplementary file 1 — Table S1: The Japanese version of the HidroQoL (HidroQoL‐J). [file JDE-53-210-s001.docx]

Table S1. The Japanese version of the HidroQoL (HidroQoL-J)

この質問票の項目は，今日を含むこの7日間に，あなたの生活が過度の発汗症状（多汗症）によってどのような影響を受けたかを尋ねるものです。各項目に対して1つだけ選択してください。項目が該当しない場合は，「いいえ，全くない」を選択してください。

| **No** |  | **非常に** | **少し** | **いいえ，**  **全くない** |
| --- | --- | --- | --- | --- |
| **領域1: 日常生活活動** | |  |  |  |
| 1 | 服を選ぶ時に影響がある | 2 | 1 | 0 |
| 2 | 身体活動に影響がある | 2 | 1 | 0 |
| 3 | 趣味に影響がある | 2 | 1 | 0 |
| 4 | 仕事に影響がある | 2 | 1 | 0 |
| 5 | 自分のコンディションを整えるためにやるべきことが増えることが気がかりだ | 2 | 1 | 0 |
| 6 | 休暇に影響がある（例：計画，行動） | 2 | 1 | 0 |
| **領域2: 心理社会的生活** | |  |  |  |
| 7 | 緊張する | 2 | 1 | 0 |
| 8 | 恥ずかしい思いをする | 2 | 1 | 0 |
| 9 | イライラする | 2 | 1 | 0 |
| 10 | 身体的な愛情表現が苦手である（例：ハグ） | 2 | 1 | 0 |
| 11 | 汗をかくことが気がかりである | 2 | 1 | 0 |
| 12 | 将来の健康が心配である | 2 | 1 | 0 |
| 13 | 周りの人の反応が心配である | 2 | 1 | 0 |
| 14 | 何らかのものに汗をつけるのが心配である | 2 | 1 | 0 |
| 15 | 新しい出会いを避ける | 2 | 1 | 0 |
| 16 | 人前で話すことを避ける（例：発表） | 2 | 1 | 0 |
| 17 | 見た目に影響がある | 2 | 1 | 0 |
| 18 | 性生活に影響がある | 2 | 1 | 0 |
